# Association of Glutathione Transferase M1, T1, P1 and A1 Gene Polymorphism and Susceptibility to IgA Vasculitis

**DOI:** 10.3390/ijms25147777

**Published:** 2024-07-16

**Authors:** Ana Juras, Kristina Crkvenac Gornik, Martina Held, Mario Sestan, Daniel Turudic, Matej Sapina, Sasa Srsen, Sanda Huljev Frkovic, Marijan Frkovic, Alenka Gagro, Marija Jelusic

**Affiliations:** 1 Department of Laboratory Diagnostics, University of Zagreb School of Medicine, University Hospital Centre Zagreb, 10 000 Zagreb, Croatia; ajuras@kbc-zagreb.hr (A.J.); kcrkvena@kbc-zagreb.hr (K.C.G.); 2 Department of Paediatrics, University of Zagreb School of Medicine, University Hospital Centre Zagreb, 10 000 Zagreb, Croatia; martina.held@mef.hr (M.H.); mario.sestan@gmail.com (M.S.); danielturudic@gmail.com (D.T.); sanda.huljev@gmail.com (S.H.F.); mfrkovic1@gmail.com (M.F.); 3 Department of Paediatrics, University Hospital Centre Osijek, Medical Faculty Osijek, Josip Juraj Strossmayer University of Osijek, 31 000 Osijek, Croatia; sapina.matej@yahoo.com; 4 Department of Paediatrics, University of Split School of Medicine, University Hospital Centre Split, 21 000 Split, Croatia; sasasrsen@yahoo.co.uk; 5 Medical Faculty Osijek, Josip Juraj Strossmayer University of Osijek, 31 000 Osijek, Croatia; alenka.gagro@gmail.com; 6 Department of Paediatrics, Children’s Hospital Zagreb, 10 000 Zagreb, Croatia

**Keywords:** IgA vasculitis, glutathione S-transferases genes, oxidative stress

## Abstract

Endothelial cell injury is a hallmark of IgA vasculitis (IgAV), possibly associated with various factors, including oxidative stress. Certain single nucleotide polymorphisms (SNPs) of glutathione S-transferases (*GST*) genes have been shown to increase susceptibility to oxidative stress. The objective of our study was to evaluate the gene polymorphisms of *GSTM1*, *GSTT1, GSTP1*, and *GSTA1* in patients with IgAV. DNA was extracted from the blood of 124 children with IgAV and 168 age-matched healthy controls. A higher frequency of the *GSTM1* null genotype was observed in patients with gastrointestinal (GI) system involvement compared to those without GI system involvement (51.5% vs. 28.6%, *p* = 0.011). Additionally, the *GSTM1* null genotype was less prevalent (30.8% vs. 69.2%, *p* = 0.032), while the *GSTP1* Val/Val genotype was significantly more prevalent in patients who developed urogenital complications (scrotal swelling) during the course of the disease (60% vs. 40%, *p* = 0.039). This study is the first to suggest an association between *GSTM1* and *GSTP1* polymorphisms and various phenotypes observed during the clinical course of IgAV in the pediatric population. However, it was performed on a national and likely single ethnic cohort, too small for definitive conclusions, so larger studies are needed to confirm this association.

## 1. Introduction

Immunoglobulin A vasculitis (IgAV), previously known as Henoch-Schönlein purpura, is a small-blood vessel vasculitis occurring predominantly in childhood [1,2]. Its most prominent sign is skin manifestations in the form of non-thrombocytopenic palpable purpura or petechiae usually distributed on the lower extremities. Although skin involvement is the most typical feature of the disease, IgAV is often associated with systemic manifestations, including gastrointestinal pain and bleeding, arthralgia, and/or arthritis and glomerulonephritis [2,3]. The etiology of IgAV remains to be clearly defined but is thought to be multifactorial, with genetic, environmental, and antigenic components [4]. 

Glutathione S-transferases (GSTs) are a superfamily of enzymes belonging to phase II enzymes, which catalyze the conjugation of many endogenous and exogenous electrophilic compounds to glutathione [5]. They are involved in the detoxification of xenobiotics and the protection of oxidative damage. In humans, GSTs are expressed in cells of all organs and tissues in addition to the cellular specific distribution of individual isoforms (e.g., GSTP in biliary tract cells). Although different isoforms can be differently expressed in the same tissue depending on tissue exposure to endobiotics and xenobiotics, high intracellular concentrations in combination with their cell-specific distribution enables the use of GSTs as biomarkers of the expected damage of a certain cell type [6,7]. 

Class mu genes are approximately 5 kb in length, and in humans all five genes are clustered together on chromosome 1p13.3 [8]. Three alleles of *GSTM1* class have been identified so far: GSTM1*A, GSTM1*B and GSTM1*0. The GSTM1*0/GSTM1*0 (*GSTM1*-null) genotype results in the deletion of the entire gene and therefore a complete lack of enzyme activity. Since no evidence of a functional difference between GSTM1*A and GSTM1*B has been reported, these two alleles are considered as a single functional phenotype. 

GSTP1 catalyzes the binding of cisplatin to glutathione so it can be converted to a water-soluble form and, hence, can be easily excreted [9]. The gene coding for the GSTP1 enzyme is found on chromosome 11q13 and is polymorphic. A non-synonymous A/G single nucleotide polymorphism at nucleotide position 313 in exon 5 results in the replacement of adenine to guanine, which results in the substitution of the amino acid isoleucine to valine at position 105. This substitution in the GSTP1 protein encoded results in decreased enzyme activity, thermal stability, and substrate specificity [10].

Because of their well-known xenobiotic metabolizing activity, genetic variations in *GST* genes have gained much attention recently. Any change in the expression of GST protein level might possibly influence an individual’s susceptibility to carcinogens and various inflammatory and chronic diseases, including rheumatic diseases [11,12,13,14].

Increased neutrophil and monocyte functions such as chemotaxis, phagocytosis, and the production of reactive oxygen species (ROSs), and an imbalance between the oxidant and antioxidant system are suggested to contribute to the pathogenesis of IgAV as well [15,16]. The defense mechanism against ROS is complex and may involve several enzymes including GSTs. 

We have recently demonstrated that increased erythrocyte glutathione S-transferase (e-GST) activity may serve as a subtle indicator of kidney function impairment in children with IgAV [17]. At disease onset, e-GST activity was significantly higher in patients who developed nephritis and remained higher after three and six months since disease onset. 

Considering the above, the aim of our study was to assess the gene polymorphisms of *GSTM1*, *GSTT1*, *GSTP1* and *GSTA1* in patients with IgAV and to investigate their possible role as a genetic component of this disease.

## 2. Results

A total number of 292 individuals (124 IgAV patients and 168 controls) were genotyped for the *GSTM1* and *GSTT1*, 289 individuals (121 IgAV patients and 168 controls) were genotyped for the *GSTA1* and 288 individuals (120 IgAV patients and 168 controls) for the *GSTP1*. All IgAV patients had skin manifestations which were severe in 6 (4.8%) patients. Arthralgia or arthritis was diagnosed in 85.5% IgAV patients, gastrointestinal (GI) involvement occurred in 41% IgAV cases, and IgA vasculitis nephritis (IgAVN) in 30.6% cases. The demographics and clinical and laboratory parameters are summarized in Table 1.

Patients with IgA vasculitis were grouped according to the dominant clinical phenotype, i.e., according to the dominant organ involvement, into the following subgroups: patients with extended purpura, patients with severe skin manifestations (bullae, ulcerations, necroses), patients with arthralgias/arthritis, patients with nephritis, patients with GI manifestations (abdominal pain, bleeding from the GI tract), patients with intestinal perforation, and patients with swelling of the scrotum (Table 2 and Table 3).

A higher frequency of the *GSTM1* null genotype was observed in IgAV patients with GI system involvement in comparison to patients without GI system involvement (51.5% vs. 28.6%, *p* = 0.011). The *GSTP1* Val/Val genotype appeared significantly more often in patients who developed urogenital complications (scrotal swelling) within the disease course (60% vs. 40%, *p* = 0.039). The same IgAV type, with scrotal swelling, shows significant differences for the *GSTM1* null genotype (30.8% vs. 69.2%, *p* = 0.032). It should be emphasized that none of the patients with scrotal edema were suffering from IgAV or nephrotic syndrome that could potentially cause scrotal swelling.

Regarding the *GSTT1* and *GSTA1* genotypes, we have not observed any difference in distribution in IgAV patients regarding their clinical phenotypes (Supplement Tables S1 and S2).

Distribution of the *GSTM1*, *GSTT1*, *GSTP1* and *GSTA1* gene polymorphisms among IgAV patients and the control group are summarized in Table 4. By the term present genotype, we considered the presence of at least one allele in the case of *GSTM1* or *GSTT1*. 

Among the 124 IgAV patients, 68 (54.8%) were found to have a *GSTM1*-null (homozygous deletion for *GSTM1*) genotype; the remaining 56 (45.2%) individuals were positive for *GSTM1* (had at least one functional *GSTM1* allele). The proportion of control group individuals with a *GSTM1* null genotype was 86 (51.2%). No significant differences were found between the IgAV group (54.8%) and the control group (51.2%) for the GSTM1 null mutation (Table 4). 

The *GSTT1* null genotype was detected in 34 (27.4%) IgAV patients and 34 (20.2%) controls. No statistically significant differences were observed between patients and the control group.

The Ile/Ile *GSTP1* genotype was observed in 63 (52.5%), the Ile/Val *GSTP1* genotype in 45 (37.5%), and the Val/Val *GSTP1* genotype in 12 (10%) IgAV patients. There was no significant difference between patients and controls. 

The frequency of the recessive alleles T/T of the *GSTA1* genotype was 17.4% in IgAV patients and 12.5% in controls, without significant differences between the two groups. The difference in *GSTA1* genotypes between the two groups was not observed using codominant, dominant, recessive, overdominant, and log-additive genetic models.

We performed logistic regression analysis to understand the association between the clinical features of IgAV and investigated *GST* gene polymorphisms (Table 5 and Table 6).

The selected variables were included in the *GSTA1* multinomial regression model: age at diagnosis, gender, a rash extended above the waist, severe skin changes (bullae, ulcerations, and necrotic lesions), arthritis and/or arthralgias, gastrointestinal involvement, nephritis, occult hemorrhage, number of relapses. Orchitis was a significant predictor between the C/C and T/T genotypes. For each unit increase in orchitis, the relative risk of orchitis being associated with C/C versus T/T genotype was 0.096 times higher, which was highly significant (*p* < 0.01). For heterozygous C/T vs. homozygous C/C genotype, the relative risk was 0.235 times higher, but the coefficient is not significant (*p* > 0.05). 

Multinomial regression could not be generated for the *GSTP1* variable due to an insufficient number of cases with the *GSTP1* wild-type Val/Val genotype [18]. 

We further investigated the potential association between polymorphisms in the analyzed *GST* genes and the response to treatment as well as the prognosis of IgAV. The analysis revealed a very weak association between the examined polymorphisms, treatment response, and prognosis. Subsequent multivariate logistic regression analysis indicated that none of the polymorphisms significantly influenced the treatment response or prognosis of IgAV (Supplement Tables S3–S5).

## 3. Discussion

Endothelial cell injury is a characteristic of IgAV, potentially linked to diverse factors like immune complex deposition, complement activation, inflammatory elements, and oxidative stress [19]. Although the roles of immune complexes, complement, and various pro-inflammatory cytokines are relatively well-established in IgAV, there is limited data on the impact of oxidative stress on the disease’s pathogenesis, specifically in relation to endothelial dysfunction [20]. Oxidative stress might contribute to the pathophysiology of autoimmune diseases, either by activating transcription factors involved in cytokine production or by engaging in oxidative post-translational modifications of proteins [21].

Members of the GST enzyme superfamily display robust antioxidant capabilities against reactive oxygen species and peroxides. Nevertheless, the ability to trigger the GST response to oxidative stress appears to be genetically influenced. Specifically, nearly all members of the GST family demonstrate genetic polymorphism, leading to either a complete absence or reduction of enzyme activity [22].

Within the *GST* genes, *GSTM1*, *GSTT1*, *GSTA1* and *GSTP1* harbor well-defined single nucleotide polymorphisms (SNPs). Homozygous deletion polymorphisms of the *GSTM1* or *GSTT1* gene, as well as the *GSTP1* Ile105 SNP and *GSTA1* T/T have been demonstrated to abolish enzyme activity and elevate susceptibility to oxidative stress. Consequently, the insufficiency in GST enzyme activity resulting from genetic polymorphisms of these genes could pose a risk factor for the development of various diseases, including glaucoma, chronic obstructive pulmonary disease, psychosis, and cancer [23,24,25,26]. To the best of our knowledge, the role of *GST* polymorphisms in the development of IgAV has not been investigated so far.

We recently published encouraging results related to the possibility of using e-GST activity as a sensitive marker of kidney function impairment in children with IgAV [17]. Namely, e-GST activity in IgAVN patients was significantly higher than in patients without nephritis and controls and remained significantly higher during the 6-month follow-up period. This prompted an investigation into whether differences exist in the distribution of specific *GST* genotypes among IgAV patients in relation to clinical phenotypes, as well as an assessment of the association between GST genotypes and the risk of developing IgAV and IgAVN. 

Our study revealed that *GST* polymorphisms do not exhibit a heightened inclination toward developing IgAV but might be associated with an elevated occurrence of gastrointestinal and urogenital manifestations. This was primarily observed for the *GSTM1* null genotype while the *GSTP1* Val/Val (*GSTP1*-low activity) genotype and *GSTA1* C/C genotype (wild type) showed certain tendencies that may be indicative.

*GSTM1* gene deletion frequencies can vary according to the ethnicity of the studied population with implications for achieving the goal of precision/personalized medicine in clinical practice. The *GSTM1* null genotypes have extensively been studied in various human populations [27]. The *GSTM1* null phenotype frequency found in the Croatian sample of healthy individuals (51.19%) is in line with the frequency observed for the European population (Austria 57.3%, France 49.0%, Italy 49.2%, Spain 55.3%) reported by other authors [28]. The best studied polymorphism, *GSTP1* Ile105Val, resulting from the substitution of the 105 Ile codon for the Val codon, most probably leads to changes in the substrate-specific thermostability and affects the conjugation activity. When compared with the *GSTP1* Ile/Ile variant, the *GSTP1* Val/Val variant was associated with reduced detoxification of epoxy diols of certain polycyclic aromatic hydrocarbons [29,30,31]. The frequencies of heterozygotes Ile/Val (43.45%) and homozygotes Val/Val (5.36%) in the Croatian sample of healthy individuals are in concordance with observed frequencies in the European population. The *GSTP1* Val/Val genotype is uncommon and exists in 5% of Caucasians [32]. In the population of white Europeans, the frequency of heterozygotes Ile/Val was between 38 and 45.7% [33]. 

While there have not been comparable studies in IgAV to date, it is noteworthy that in one of the limited studies investigating the impact of oxidative stress on the development of IgAV, heightened DNA oxidation was observed in patients with gastrointestinal involvement during the active phase of IgAV [19]. This could be attributed to the susceptibility of gastrointestinal cells to oxidative damage or the efficient repair mechanisms of these cells. Gastrointestinal involvement in IgAV may resemble the early phases of inflammatory bowel disease (IBD), both clinically and histologically [34]. Studies investigating oxidative DNA damage in IBD have demonstrated elevated levels of 8-hydroxydeoxyguanosine, a marker of oxidative DNA damage, in the affected patients. In these investigations, the levels of 8-hydroxydeoxyguanosine remained unaffected by systemic inflammation or disease activity. The findings suggest an enhanced vulnerability of gastrointestinal cells to DNA oxidation in patients with IBD, mirroring our observations in patients with gastrointestinal involvement in IgAV [34,35,36].

Concerning the link between *GST* polymorphisms and the urogenital system, some research shown that variations in *GSTM1*, *GSTT1*, and *GSTP1* are linked to male infertility [37,38,39]. This is attributed to the primary role of the enzymes encoded by *GST*s in mitigating reactive oxygen species. Consequently, these enzymes are more likely to influence the quantity, survival, and/or activity of produced sperm.

When considering *GSTA1* polymorphisms, those linked to a reduced expression of the GSTA1 enzyme may result in an accumulation of carcinogens in the body, thereby elevating cancer risk [40]. It has been observed that the expression of this enzyme rises under conditions of oxidative stress and proliferative hyperplasia related to chronic inflammation [41], but decreases in prostate intraepithelial tumors [42]. This supports the hypothesis that *GSTA1* could play a role in the development of prostate cancer. Nonetheless, the role of this enzyme has not been examined in IgAV.

Our study does not provide an explanation for why *GST* polymorphisms demonstrated an influence solely on gastrointestinal and urogenital manifestations in IgAV, without affecting other organ systems like kidneys, skin, or joints. Nevertheless, the finding regarding the absence of a link between *GST* polymorphisms and kidney involvement in IgAV is particularly surprising. This is because certain studies have indicated that the *GSTM1*-null genotype is correlated with a higher risk of developing end-stage renal disease and an increased susceptibility to oxidative stress in dialysis patients [43]. Conversely, certain studies have not identified associations between the activity e-GST (enzymes encoded by *GST* genes) and markers of systemic inflammation or kidney function [6,17,44]. 

Another potential link between *GST* gene polymorphisms and IgAV is illustrated through the case of cyclophosphamide, which is also utilized in the treatment of severe nephritis in individuals with IgAV. Indeed, studies have demonstrated a significant association between the allelic variation *GSTP1* Ile105Val and disease remissions dependent on cyclophosphamide treatment in patients with various autoimmune diseases, including vasculitis. Individuals carrying the Ile105Val SNP in at least one copy exhibited a notably higher response rate to the treatment [45]. This specific variant of *GSTP1*, characterized by a lower conjugation capacity, leads to an extended and higher therapeutic dose of cyclophosphamide, suggesting that the reduced activity of this *GSTP1* variant may underlie more effective disease treatment. In our study, we did not observe an association between polymorphisms in the analyzed *GST* genes and the response to treatment as well as the prognosis of IgAV. Additionally, the patients in this study were administered alternative immunosuppressants rather than cyclophosphamide. 

We need to recognize several limitations of this case-control study. The relatively small sample size could significantly reduce the statistical power to investigate genuine associations, so for any potential associations further larger studies are needed. The lack of an association between the investigated *GST* polymorphisms and susceptibility to IgAV was unexpected. The absence of interdependence cannot exclude the possibility of an association in another population or different ethnic group since genetic association studies are susceptible to population-specific genotype effects. Furthermore, we were not able to perform functional analyses, i.e., measuring markers of oxidative stress. Finally, we did not examine gene-gene or gene-environment interactions.

## 4. Materials and Methods

### 4.1. The Patients Selection and Study Design

A case-control study included 124 children with IgAV from three Croatian tertiary centers for pediatric rheumatology in the period between 2019 and 2022. Patients were enrolled in the study upon satisfying the criteria set by the European League Against Rheumatism (EULAR)/Pediatric Rheumatology International Trials Organization (PRINTO)/Pediatric Rheumatology European Society (PRES) [46]. Clinical data and laboratory parameters were collected from medical records. Four IgAV patients were excluded from *GSTP1* statistical analysis due to amplification failure. A total of 168 age- and sex-matched individuals without any history of autoimmune diseases were selected as a control group. All the participants and/or their parents or their legal guardians provided written informed consent and the study was carried out with the principles of the Declaration of Helsinki.

### 4.2. GST Genotyping

3 mL of peripheral venous blood was collected from participants into sterile EDTA-containing tubes. Immediately after collection, the whole blood was stored at +4 °C until use. Whole EDTA blood was used for genomic DNA extraction using QIAamp DNA Mini Kit kit (Qiagen, Inc., Chatsworth, CA, USA) according to the manufacturer’s instructions. For assessing the presence of amplified PCR products of *GSTM1* and *GSTT1* multiplex PCR was performed. The housekeeping gene *CYP1A1* was applied as an internal control [47]. Primers used were: *GSTM1* forward: 5′-GAACTCCCTGAAAAGCTAAAGC-3′ and *GSTM1* reverse: 5′-GTTGGGCTCAAATATA CGGTGG-3′. Exon 7 of the *CYP1A1* gene was co-amplified and used as an internal control using the following primers: *CYP1A1* forward: 5′-GAACTGCCACTT CAGCTGTCT-3′ and *CYP1A1* reverse: 5′-CAGCTGCATTTGGAAGTGCTC-3′. The primers used were *GSTT1*-forward: 5′-TTCCTTACTGGTCCTCACATCTC-3′ and *GSTT1*-reverse: 5′-TCACGGGATCAT GGCCAGCA-3′ [47]. The assay does not distinguish heterozygous from homozygous wild type genotypes. Therefore, it notes only the presence (at least one allele present, homozygote or heterozygote—*GSTM1*-active genotype, *GSTT1*-active genotype) or the absence (complete deletion of both alleles, homozygote—*GSTM1*-null genotype, *GSTT1*-null genotype) of the specific genotype. PCR was carried out in DNA thermal cycler (Mastercycler PCR, Eppendorf, Hamburg, Germany). The presence of 215 bp band indicated the presence of amplified *GSTM1* gene. The presence of 480 bp band indicated the presence of the amplified *GSTT1* gene. PCR products were separated on a 2.5% agarose gel at 150 V for 10 min and stained with ethidium bromide. Single nucleotide polymorphisms of *GSTP1* were analyzed using the PCR–RFLP method by Harries et al. [48]. The *GSTA1* polymorphism was determined by PCR–restriction fragment length polymorphism according to Ping et al. [49]. The primers used were *GSTA1* forward: 5′-TGT TGA TTG TTT GCC TGA AAT T-3′ and *GSTA1* reverse: 5′-GTT AAA CGC TGT CAC CCG TCC T-3′. The PCR was carried out in PCR thermal cycler (Eppendorf Mastercycler, Hamburg, Germany). PCR products were separated on a 2.5% agarose gel at 150 V for 10 min and stained with ethidium bromide. The presence of 176 bp band indicated the presence of the amplified *GSTP1* gene. The presence of 481 bp band indicated the presence of the amplified *GSTA1* gene. The amplification products (20 µL) were digested by 2U of restriction endonuclease Alw26I at 37 °C for 16 h. The restriction products were separated on a 2.5% agarose gel. The restriction site resulting in two fragments (91 bp and 85 bp) indicated a *GSTP1* Val/Val (*GSTP1*-low activity) genotype. The presence of three fragments (176 bp, 91 bp and 85 bp) was indicative for *GSTP1* Ile/Val genotype, while the presence of one 176 bp band indicated a *GSTP1* Ile/Ile genotype (referent genotype). For the *GSTA1* gene, the restriction site resulting in two fragments (385 bp and 96 bp) indicated a *GSTA1* T/T (*GSTA1*-low activity) genotype, while the presence of one 481 bp band indicated a *GSTA1* C/C genotype (referent genotype). The presence of three fragments (481 bp, 385 bp and 96 bp) was indicative of a *GSTA1* C/T genotype.

### 4.3. Statistical Analysis

Numerical data were described as mean and standard deviation (SD) or median [interquartile range (IQR)], while categorical variables were presented as whole numbers (N) with percentages (%). The normality of the data distribution was assessed using the Shapiro-Wilk test. Differences in the categorical variables between groups were assessed using χ^2^ and Fisher exact test and among the numerical ones using the Mann-Whitney U test. Binomial and multinomial logistic regression analyses were performed to investigate the association between the *GSTM1, GSTT1, GSTP1*, and *GSTA1* gene polymorphisms in several IgAV and control group phenotypes. Kendall’s tau correlation coefficient was used to investigate the correlation between gene polymorphisms and treatment (glucocorticoids, immunosuppressants). Binomial and multinomial logistic regression models were generated in R ver 4.2.3. Binomial logistic regression models were generated using the generalized linear models (glm) function embedded within the R base packages. The multinomial logistic regression model was generated using the net [50] package. Relative risks between the *GSTA1* phenotypes were calculated using the Stargazer R package [51]. The level of statistical significance was determined with *p* < 0.05.

## 5. Conclusions

In conclusion, the etiology of IgAV is intricate, encompassing genetic and environmental factors. Our data suggest that the *GSTM1*, *GSTT1*, *GSTA1* and *GSTP1* genotypes are not associated with susceptibility to IgAV in the population of Croatia nor with the response to treatment or the prognosis of the disease. However, when considering IgAV type with GI and urological complications separately, *GSTM1* and *GSTP1* genotyping was different from healthy subjects. Collectively, these findings suggest that individuals with IgAV who exhibit gastrointestinal and urogenital involvement may experience changes in metabolic pathways related to the elimination of reactive oxygen species. Considering the genetic polymorphism and relation of studied genes with autoimmune disorders, the results may be valued as a contribution to the literature. However, the study was conducted on a national and likely single ethnic cohort, which was too small to draw definitive conclusions. Therefore, larger studies are necessary to validate this association.

## Figures and Tables

**Table 1:** Demographic data, clinical features and laboratory findings in patients with IgAV.

Characteristics of the Patients	N = 124	% of the Cohort		
Demographics			Laboratory Parameters	
F	67	54%	ESR (mm/h)	21.5 ± 16.3
M	57	46%	CRP (mg/L)	14.6 ± 19.5
age	6.3 (4.3–8.1)		leukocytes (10^9^/L)	10.7 ± 3.4
height (cm)	126 (109–137)		erythrocytes (10^12^/L)	4.7 ± 0.4
weight (kg)	24.1 (17.8–34.8)		hemoglobin (g/L)	124.7 ± 10.5
BMI (kg/m^2^)	15.9 (14.4–19.8)		hematocrit	0.36 ± 0.03
**Clinics**			platelets (10^9^/L)	353 ± 90.9
Skin manifestations	124	100%	creatinine (µmol/L)	36.9 ± 12.2
purpuric rash	124	100%	urea (mmol/L)	4.3 ± 1.1
rash extended above waist	57	46%	fibrinogen (g/L)	3.53 ± 1.04
recurrent rash	17	13.7%	D-dimer (µg/L)	4.4 ± 6.3
bullae, ulcerations, and necrotic lesions	6	4.8%	PT	1.01 ± 0.15
Arthritis and/or arthralgias	106	85.5%	aPTT (s)	24.8 ± 4.7
Gastrointestinal involvement	51	41%	ferritin (ng/mL)	68.8 ± 43.2
abdominal pain	37	29.8%	uACR (mg/mmol)	26.9 ± 94.8
positive FOBT	29	23.4%	24-h proteinuria (g/dU)	0.24 ± 0.6
bowel perforation	1	0.8%	E/mm^3^ (urine spot test) (%)	30 (24.2%)
intussusception	3	2.4%	eGFR (mL/min/1.73 m^2^)	134.4 ± 29.7
Nephritis	38	30.6%	total proteins (g/L)	68.6 ± 6.3
Orchitis	10	17.5% ^#^	serum albumin (g/L)	39.3 ± 4.7
Disease relapse	30	24.2%	IgA (g/L)	1.8 ± 0.8
**Treatment**			IgG (g/L)	10.5 ± 2.7
NSAIDs	88	71%	IgM (g/L)	1.1 ± 1.1
glucocorticoids	54	43.5%	C_3_ (g/L)	1.32 ± 0.24
ACE inhibitors	16	12.9%	C_4_ (g/L)	0.26 ± 0.1
immunosuppressants	9	7.3%	CH50 (%)	94 ± 22.7
**Outcome of nephritis**			fecal calprotectin (µg/g)	167.3 ± 371.3
A	28	73.7%		
B	10	26.3%		
C	0	0%		
D	0	0%		

Legend: Data are presented as a whole number (%) or as mean ± standard deviation (SD) or median (IQR); F: female; M: male; BMI: body mass index; ESR: erythrocyte sedimentation rate; CRP: C-reactive protein; PT: prothrombin time; aPTT: activated partial thromboplastin time; uACR: urine albumine to creatinine ratio; E/mm^3^: erythrocytes per mm^3^ of urine; eGFR: estimated glomerular filtration rate; IgA: immunoglobulin A; IgG: immunoglobulin G; IgM: immunoglobulin M; C_3_—complement component; C_3_, C_4_—complement component; CH50—total complement activity; FOBT: fecal occult blood test; NSAIDs: non-steroidal anti-inflammatory drugs; ACE: angiotensin converting enzyme; A: normal on physical examination, with normal urine or microhematuria and normal renal function; B: normal on physical examination, with proteinuria <1 g per day or <40 mg/h per m^2^, urine albumine to creatinine ratio <200 mg/mmol; C: proteinuria >1 g per day or >40 mg/h/m^2^ and/or hypertension, glomerular filtration rate >60 mL/min/1.73 m^2^; D: renal insufficiency (estimated glomerular filtration rate <60 mL/min/1.73 m^2^ or end stage renal disease (estimated glomerular filtration rate <15 mL/min/1.73 m^2^) or death; ^#^ applicable only for boys.

**Table 2:** The distribution of particular genotypes for the *GSTM1* polymorphism in IgAV patients regarding clinical phenotypes.

Clinical Feature		*GSTM1* Null N = 68	*GSTM1* Present N = 56	*p* Value
**rash extended above waist**	yes	32 (47.1%)	25 (44.6%)	0.788 ^a^
	no	36 (52.9%)	31 (55.4%)	
recurrent rash	yes	10 (14.7%)	7 (12.5%)	0.722 ^a^
	no	58 (85.3%)	49 (87.5%)	
bullae, ulcerations, and necrotic lesions	yes	3 (4.4%)	3 (5.4%)	>0.999 ^b^
	no	65 (95.6%)	53 (94.6%)	
arthritis and/or arthralgias	yes	59 (86.8%)	47 (83.9%)	0.849 ^a^
	no	9 (13.2%)	9 (16.1%)	
gastrointestinal involvement	yes	35 (51.5%)	16 (28.6%)	0.011 ^a^*
	no	33 (48.5%)	40 (71.4%)	
bowel perforation	yes	0 (0%)	1 (1.8%)	0.452 ^b^
	no	68 (100%)	55 (98.2%)	
nephritis	yes	25 (36.7%)	13 (23.2%)	0.106 ^a^
	no	43 (63.2%)	43 (76.8%)	
orchitis ^#^	yes	2 (6.4%)	8 (30.8%)	0.032 ^b^*
	no	29 (93.6%)	18 (69.2%)	
disease relapse	yes	20 (29.4%)	10 (17.8%)	0.138 ^a^
	no	48 (70.6%)	46 (82.2%)	

Data are presented as a whole number (%); ^#^ applicable only for boys; ^a^ chi-square test; ^b^ Fisher exact test; * *p* < 0.05.

**Table 3:** The distribution of particular genotypes for the *GSTP1* polymorphism in IgAV patients regarding clinical phenotypes.

Clinical Feature		*GSTP1* N = 120			*p* Value
		Ile/Ile N = 63	Ile/Val N = 45	Val/Val N = 12	
**rash extended above waist**	**yes**	31 (49.2%)	17 (37.8%)	8 (66.7%)	0.172 ^a^
	no	32 (50.8%)	28 (62.2%)	4 (33.3%)	
recurrent rash	yes	11 (17.5%)	4 (8.9%)	2 (16.7%)	0.472 ^b^
	no	52 (82.5%)	41 (91.1%)	10 (83.3%)	
bullae, ulcerations, and necrotic lesions	yes	3 (4.8%)	2 (4.4%)	0 (0%)	>0.999 ^b^
	no	60 (95.2%)	43 (95.6%)	12 (100%)	
arthritis and/or arthralgias	yes	54 (85.7%)	39 (86.7%)	9 (75%)	0.587 ^a^
	no	9 (14.3%)	6 (13.3%)	3 (25%)	
gastrointestinal involvement	yes	23 (36.5%)	19 (42.2%)	7 (58.3%)	0.386 ^a^
	no	40 (63.5%)	26 (57.8%)	5 (41.7%)	
bowel perforation	yes	0 (0%)	0 (0%)	1 (8.3%)	0.100 ^b^
	no	63 (100%)	45 (100%)	11 (91.7%)	
nephritis	yes	19 (30.2%)	15 (33.3%)	4 (33.3%)	0.933 ^a^
	no	44 (69.8%)	30 (66.7%)	8 (66.7%)	
Orchitis ^#^	yes	6 (18.2%)	1 (5.5%)	3 (60%)	0.039 ^b^*
	no	27 (81.8%)	17 (94.5%)	2 (40%)	
disease relapse	yes	16 (25.4%)	10 (22.2%)	3 (25%)	0.928 ^a^
	no	47 (74.6%)	35 (77.8%)	9 (75%)	

Data are presented as a whole number (%); ^#^ applicable only for boys; ^a^ chi-square test; ^b^ Fisher exact test; * *p* < 0.05.

**Table 4:** *GST* genotypes and risk of developing IgAV.

Genotype	Control Group N = 168	IgAV Patients N = 124	OR (95%) CI	*p* Value
***GSTM1***				
present	82 (48.8%)	56 (45.2%)	0.727–1.844	0.537
null	86 (51.2%)	68 (54.8%)		
***GSTT1***				
present	134 (79.8%)	90 (72.6%)	0.863–2.568	0.151
null	34 (20.2%)	34 (27.4%)		
***GSTP1***				
Ile/Ile	86 (51.2%)	63 (52.5%)	0.799–4.819	0.259
Ile/Val	73 (43.5%)	45 (37.5%)		
Val/Val	9 (5.3%)	12 (10%)		
***GSTA1***				
codominant				
C/C	59 (35.1%)	47 (38.8%)	0.763–2.833	0.292
C/T	88 (52.4%)	53 (43.8%)		
T/T	21 (12.5%)	21 (17.4%)		
dominant				
C/C	59 (35.1%)	47 (38.8%)	0.525–1.382	0.517
C/T-T/T	109 (64.9%)	74 (61.2%9		
recessive				
C/C-C/T	147 (87.5%)	100 (82.6%)	0.762–2.833	0.251
T/T	21 (12.5%)	21 (17.4%)		
overdominant				
C/C-T/T	80 (47.6%)	68 (56.2%)	0.443–1.133	0.149
C/T	88 (52.4%)	53 (43.8%)		
log-aditive				
0,1,2	168 (58.1%)	121 (41.9%)	NA	0.889

**Table 5:** Binomial logistic regression analysis of association between *GSTM1* and *GSTT1* polymorphisms and the clinical features of IgAV.

Clinical Feature	Genotype	SE	Z-Value	OR	95% CI	*p* *
age	***GSTM1***	0.001	−1.513	0.999	0.998–1.000	0.130
	***GSTT1***	0.001	0.556	1.000	0.999–1.002	0.578
gender	***GSTM1***	0.407	−0.988	0.668	0.296–1.475	0.323
	***GSTT1***	0.440	−0.995	0.645	0.267–1.516	0.320
rash extended above waist	***GSTM1***	0.432	0.643	1.32	0.568–3.118	0.520
	***GSTT1***	0.473	0.445	18.966	0.490–3.175	0.656
recurrent rash	***GSTM1***	0.847	0.391	1.393	0.248–7.396	0.695
	***GSTT1***	0.875	−0.780	0.505	0.078–2.654	0.435
bullae, ulcerations, and necrotic lesions	***GSTM1***	0.939	0.051	1.049	0.155–7.065	0.959
	***GSTT1***	0.925	−0.424	1.234	0.116–5.287	0.672
arthritis and/or arthralgias	***GSTM1***	0.582	−1.064	0.538	0.167–1.681	0.287
	***GSTT1***	0.698	−0.700	0.613	0.129–2.183	0.484
gastrointestinal involvement	***GSTM1***	0.612	−2.117	0.274	0.075–0.857	**0.034**
	***GSTT1***	0.642	0.370	1.268	0.379–4.934	0.711
nephritis	***GSTM1***	0.502	−0.633	0.727	0.266–1.941	0.526
	***GSTT1***	0.578	0.379	1.245	0.419–4.187	0.704
orchitis ^#^	***GSTM1***	0.874	−2.215	0.161	0.022–0.728	**0.027**
	***GSTT1***	0.867	−0.121	0.900	0.123–4.344	0.903
Intercept	***GSTM1***	2.138	1.249	8.574	0.132–645.067	0.3149
	***GSTT1***	2.944	1.005	0.6756	0.212–544.054	0.212

Legend: SE: standard error; OR: odds ratio; CI: confidence interval; ^#^ applicable only for boys. Statistical significance was set to * *p* < 0.05 and bolded.

**Table 6:** Multinomial logistic regression for the association between different *GSTA1* polymorphisms and the clinical features of IgAV. The wild-type genotype (C/C) was selected as the reference category.

	Dependent Variable			
	C/T Genotype		T/T Genotype	
Clinical Feature	Relative Risk	95% CI	Relative Risk	95% CI
age	1.000	(0.999, 1.002)	0.998	(0.996, 1.000)
gender	1.872	(−0.039, 3.782)	1.048	(−0.382, 2.477)
rash extended above waist	2.569	(1.366, 3.771)	2.050	(0.681, 3.419)
recurrent rash	0.350	(−1.486, 2.186)	1.065	(−1.247, 3.377)
bullae, ulcerations, and necrotic lesions	0.977	(−1.568, 3.523)	1.852	(−0.915, 4.619)
arthritis and/or arthralgias	2.505	(0.812, 4.198)	1.458	(−0.505, 3.420)
gastrointestinal involvement	2.530	(0.893, 4.166)	5.287	(3.457, 7.11)
nephritis	1.072	(−0.417, 2.560)	2.794	(1.161, 4.426)
orchitis ^#^	0.235	(−1.650, 2.120)	0.096 *	(−1.850, 2.041)
Constant	1.872	(−0.039, 3.782)	1.048	(−0.382, 2.477)
Akaike Inf. Criterion	146.968		146.968	

Legend: CI: confidence interval; ^#^ applicable only for boys. Note: * *p* < 0.05.

## Data Availability

The original contributions presented in the study are included in the article, further inquiries can be directed to the corresponding author.

## Supplementary Materials

The following supporting information can be downloaded at: https://www.mdpi.com/article/10.3390/ijms25147777/s1.
