# Supplementary material for: Association of Glutathione Transferase M1, T1, P1 and A1 Gene Polymorphism and Susceptibility to IgA Vasculitis
Source: Int J Mol Sci. 2024 Jul 16;25(14):7777. doi: 10.3390/ijms25147777 (PMC11277070; doi:10.3390/ijms25147777)
Supplement: Supplementary file 1 [file ijms-25-07777-s001.zip › ijms-2877128-supplementary.pdf]

Supplement Table S1. The distribution of particular genotypes for the *GSTT1* polymorphism in IgAV patients regarding clinical phenotypes.

clinical feature		*GSTT1* null N=34	*GSTT1* present N=90	p value
rash extended above waist	yes	12 (35.3%)	44 (48.9%)	0.248[a]
	no	22 (64.7%)	46 (51.1%)	
recurrent rash	yes	4 (11.8%)	13 (14.4%)	0.971[a]
	no	30 (88.2%)	77 (85.6%)	
bullae, ulcerations, and necrotic lesions	yes	2 (5.9%)	4 (4.4%)	1.00[b]
	no	32 (94.1%)	86 (95.6%)	
arthritis and/or arthralgias	yes	31 (91.2%)	75 (83.3%)	0.412[a]
	no	3 (8.8%)	15 (16.7%)	
gastrointestinal involvement	yes	11 (32.4%)	40 (44.4%)	0.367[a*]
	no	23 (67.6%)	50 (55.6%)	
bowel perforation	yes	0 (0%)	1 (1.1%)	1.00[b]
	no	34 (100%)	89 (98.9%)	
nephritis	yes	10 (29.4%)	27 (30%)	1.00[a]
	no	24 (70.6%)	63 (70%)	
orchitis[#]	yes	2 (16.7%)	8 (18.2%)	1.00[b*]
	no	10 (83.3%)	36 (81.8%)	
disease relapse	yes	7 (20.6%)	22 (24.4%)	0.577[a]
	no	27 (79.4%)	68 (75.6%)	

Data are presented as a whole number (%); #applicable only for boys; [a]chi-square test; [b]Fisher exact test; *p<0.05.

Supplement Table S2. The distribution of particular genotypes for the *GSTA1* polymorphism in IgAV patients regarding clinical phenotypes.

clinical feature		GSTA1 N=121			p value
		C/C N=47	C/T N=53	T/T N=21	
rash extended above waist	yes	20 (42.6%)	24 (45.3%)	11 (52.4%)	0.753[a]
	no	27 (57.4%)	29 (54.7%)	10 (47.6%)	
recurrent rash	yes	4 (8.7%)	10 (18.9%)	3 (14.3%)	0.351[b]
	no	42 (91.3%)	43 (81.1%)	18 (85.7%)	
bullae, ulcerations, and necrotic lesions	yes	2 (4.3%)	2 (3.8%)	1 (4.8%)	0.980[b]
	no	45 (95.7%)	51 (96.2%)	20 (95.2%)	
arthritis and/or arthralgias	yes	40 (85.1%)	45 (84.9%)	18 (85.7%)	0.996[a]
	no	7 (14.9%)	8 (15.1%)	3 (14.3%)	
gastrointestinal involvement	yes	15 (32.6%)	25 (47.2%)	9 (42.9%)	0.332[a]
	no	31 (67.4%)	28 (52.8%)	12 (57.1%)	
bowel perforation	yes	1 (2.1%)	0 (0%)	0 (0%)	0.444[b]
	no	46 (97.9%)	53 (100%)	21 (100%)	
nephritis	yes	14 (29.8%)	14 (26.4%)	9 (42.9%)	0.379[a]
	no	33 (70.2%)	39 (73.6%)	12 (57.1%)	
orchitis[#]	yes	1 (5.6%)	4 (18.2%)	5 (33.3%)	0.120[b*]
	no	17 (94.4%)	18 (81.8%)	10 (66.7%)	
disease relapse	yes	9 (19.1%)	16 (30.2%)	4 (19.1%)	0.579[a]
	no	38 (80.9%)	37 (69.8%)	17 (80.9%)	

Data are presented as a whole number (%); #applicable only for boys; [a]chi-square test; [b]Fisher exact test; *p<0.05.

Supplement Table S3. Logistic regression analysis of associaton between different *GST* polymorphisms and response to glucocorticoids.

Genotype	SE	Z-value	p value
GSTT1	0.35562	0.43264	0.411
GSTM1	-0.48554	0.37781	0.199
GSTA1	0.29412	0.26580	0.268
GSTP1	0.08124	0.28596	0.776

Supplement Table S4. Logistic regression analysis of associaton between different *GST* polymorphisms and response to immunosupresives.

Genotype	SE	Z-value	p value
GSTT1	0.3033	0.8517	0.7218
GSTM1	-1.3577	0.8541	0.1119
GSTA1	0.8195	0.5220	0.1164
GSTP1	-0.7056	0.6518	0.2791

Supplement Table S5. Logistic regression analysis of associaton between different *GST* polymorphisms and prognosis.

Genotype	SE	Z-value	p value
GSTT1	-0.25585	0.74714	0.7320
GSTM1	-1.31825	0.81858	0.1073
GSTA1	-0.08453	0.49271	0.8638
GSTP1	0.57121	0.50529	0.2583
